# Process of developing Country Cooperation Strategy in Tanzania, as an effective tool for aligning WHO’s support to the member state in achieving health and health-related sustainable development goal

**DOI:** 10.11604/pamj.supp.2023.45.1.39584

**Published:** 2023-06-07

**Authors:** Sisay Gashu Tegegne, Tigest Ketsela Mengestu, Katayama Francisco, Caroline Bollars, Happy Kapanga, Fedjo Tefoyet Galbert, Ghirmay Andemichael, Christine Musanhu, Mapunda Maxmillan, Kileo Neema, Grace Saguti, Jiri Phyllis, Peter Nsubuga, Yoti Zablon

**Affiliations:** 1 World Health Organization Country Office, Dar es Salaam, Tanzania,; 2 World Health Organization African Region, Brazzaville, Congo,; 3 World Health Organization European Regional Office, Copenhagen, Denmark,; 4 Ministry of Health, Dodoma, Tanzania,; 5 Global Public Health Solutions, Atlanta, Georgia, USA

## Abstract

**Introduction:**

an organization’s long-term success and relevance are linked with compelling strategic development. To that end, the country office of WHO in the United Republic of Tanzania, in collaboration with stakeholders, developed a 6-year Country Cooperation Strategy (CCS), 2022-2027. This paper describes the various steps taken in developing the CCS for the United Republic of Tanzania.

**Methods:**

we reviewed the global guideline for the development of CCS. In addition, we analysed documents on the national health sector strategic plan, the 13th Global Program of Work for WHO (GPW13), and the Sustainable Development Goal (SDG). We also reviewed data from routine HMIS, the Global Burden of Disease (GBD), and assessment results of the UN on the status of SDGs through the Common Country Assessment (CCA).

**Results:**

the performance on the overall Universal Health Coverage (UHC) effective coverage index, on a scale of 0-100, for Tanzania improved from 45.2 in 2010 to 55.2 in 2019. Strengthening health systems, protecting communities against public health emergencies, reducing or controlling exposure of individuals to risk factors, and better health governance, leadership, and accountability were the identified priorities for the CCS.

**Conclusion:**

the process of alignment of the CCS document with the national and global strategic goals would help the WHO to support and lead the country’s effort towards achieving health-related SDGs. We believe the process we employed will lead to having detailed operational plans for implementation for achieving SDG targets.

**Keywords:**

Country cooperation strategy (CCS), sustainable development goal (SDG), strategic document, 13th global program of work (GPW13), health sector strategy, stakeholders, Tanzania

## Introduction

A literature review on the role of strategic planning by Kraus *et al*. emphasizes its importance in the predictability of possible future scenarios and variations as well as a central instrument for the strategic management of goals and visions of an institution changing [1]. Any organization´s long-term success and relevance are linked with a compelling strategic development process; various studies on this line demonstrated that comprehensive strategic planning contributes towards better organizational performance [2-4]. Strategic planning is a practice of management that links planning exercise with implementation, an ongoing process. It is a tool for accountability and compliance [5].

The United Nations (UN) Sustainable Development Goal (SDG) 3 is among the 17 goals that member states agreed to achieve by 2030. Goal 3 is to ensure healthy lives and promote well-being for all ages. Countries should have a strategic approach and plan to address the 13 targets under this goal. Clear obligations and responsibilities for all member states call for developing a strategic approach to be on track with targets. All countries are expected to take ownership and establish a strategic framework in line with the SDGs, to be on track with the goals [6,7]. WHO developed the thirteenth General Program of Work (GPW13) as a strategic approach to lead member states to attain health-related SDGs. This strategic document guides member states on high-level outcomes and outputs each country focuses on to achieve country-specific targets. It also provides a framework for countries to monitor global targets in health [8,9].

The Tanzania Development Vision 2025 states that attaining primary health care is one of the means to achieve a high-quality livelihood for the nation. In addition, the various national policies, such as the fifth Health Sector Strategic Plan 2016-2025 (HSSP V), sustainable and rapid reduction of maternal, newborn and child deaths and overall improved access to related quality services all highlighted the government priorities in health and areas of collaboration with stakeholders in strategic plans and implementations [10-12]. The UN developed a framework as one country team to support the government´s effort to achieve the SDG targets. The common country assessment of the UN support for the country to achieve SDGs highlighted non-communicable disease, quality of care, and challenges in the health system are roadblocks on the way to achieving health-related SDGs [13]. WHO leads the health efforts of the UN in the UN framework and coordinates health partners. To that end, a Country Cooperation Strategy (CCS) is the WHO´s corporate framework strategy in response to country needs and priorities in line with GPW13. It addresses health and health-related SDGs [14].

The current CCS for the United Republic of Tanzania was developed in line with the challenges identified and the best practices that were recorded in the country. It is the WHO´s support at the three levels of the organization to support the country to be on track with health-related SDGs with the principle of leaving no one behind. This paper describes the various steps taken in developing the CCS for Tanzania for 2022-2027. It also describes the actions to implement the strategic priorities successfully.

## Methods

**Figure 1:**
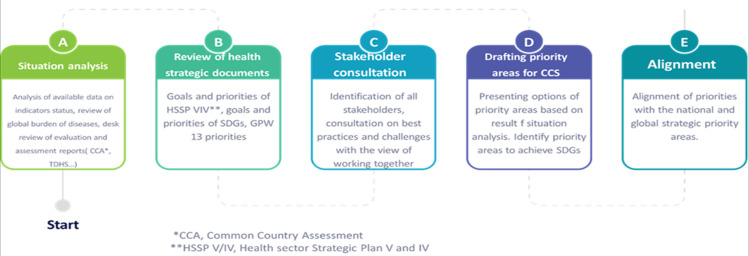
flow chart to scan environmental analysis of the various strategic documents in the country: process of developing priority areas for ccs to make a big impacting achieving SDGs

**Team to work on the preparation of the strategic document:** at the beginning of October 2021, a working group comprising the WHO country office, the Ministry of Health and representatives of the WHO Regional Office for Africa and WHO headquarters was established to lead the process of developing the CCS. The group set terms of reference and a timeline of 6 months for completing the work, and a regular weekly meeting was scheduled to monitor the progress. We started the consultation process with the WHO Regional Office and headquarters supporting the process. The review was focused on the various steps and processes we should employ, lessons learned from other countries that went through the same process of developing similar documents and most notably on the focus of alignment with available national and global priority areas. We reviewed the global guideline for developing the country cooperation strategy, 2020, as a checklist and guide for alignment with national strategic and development plans and the priorities of the United Nations Sustainable Development Framework (UNSDCF) for Tanzania. We used an approach to learn the internal and external factors in achieving the targets. This approach included a situation analysis, a review of national and global strategic documents, stakeholder consultation, and an alignment of priorities (Figure 1). The result of these assessments was used to formulate recommendations, including focused priority areas.

**Review of health information and strategic plans towards sustainable development goals:** we reviewed various documents in-depth to learn the status of targets and priorities in the national health sector development plan toward achieving the health and health-related SDGs. We reviewed the global burden of disease data and information for Tanzania to observe trends. We analysed what causes the most deaths and the risk factors that drive the most deaths and disabilities in Tanzania [15,16]. We also reviewed the paper published by the global burden of disease collaborators on measuring universal health coverage based on an index of adequate coverage of health services. This method used three steps to determine adequate health coverage for each country. First, an analysis of the proxy coverage of selected 23 indicators (Table 1, Table 1 (suite), Table 1 (suite 1), Table 1 (suite 2), Table 1 (suite 3)), second a calculation of the fraction of potential health gains associated with each indicator. Third, the overall Universal Health Coverage (UHC) effective coverage index was constructed by weighting each coverage indicator of the 23 selected indicators relative to its health gains fractions. This tool was effective in strategic planning in priority setting [17]. We used the routine health information reported through DHIS2/HMIS to see the achievements in various indicators. Indicators on reproductive, maternal, newborn, child, and adolescent health (RMNCAH), infectious disease control and prevention, non-communicable diseases (NCDs) and health systems were used to learn the strength and the gap in health delivery to the community. We reviewed the trend of the indicators over time.

**Table 1:** details of 23 effective coverage indicators included in the universal health coverage effective coverage index, by health service type, 2019 (source lancet 2020, ensuring universal health coverage, 1990-2019: by GBD universal health coverage collaborators)

	Effective coverage indicator	Metric	Effective coverage indicator measurement	
			**Numerator**	**Denominator**
**Reproductive and newborn**				
Promotion	Met need for family planning with modern contraception	Coverage	Females aged 15-49 years with demand for family planning met with modern contraception	Females aged 15-49 years with demand for family planning
Prevention; treatment, communicable diseases and MNCH	Antenatal, peripartum, and postnatal care for newborn babies	Early neonatal mortality rate	All-cause deaths during the first 7 days of life	Population of early neonates
Prevention; treatment, communicable diseases and MNCH	Antenatal, peripartum, and postnatal care for mothers	Maternal mortality ratio	Deaths due to maternal disorders for females aged 10-54 years	Livebirths among females aged 10-54 years
**Children younger than 5 years**				
Prevention	DTP3 vaccine coverage	Coverage	Receipt of three doses of DTP vaccine among children aged 12-23 months	Children aged 12-23 months
Prevention	MCV1 coverage	Coverage	Receipt of MCV1 among children aged 12-23 months	Children aged 12-23 months
Treatment, communicable diseases and MNCH	LRI treatment	MIR	Mortality from LRIs for children younger than 5 years	Incidence of LRIs for children younger than 5 years

GBD= global burden of disease, MNCH= maternal, neonatal, and child health, DTP3= diphtheria-tetanus-pertussis vaccine, 3 doses, MCV1= measles-containing-vaccine, 1 dose, LRI= lower respiratory infection, MIR= mortality-to-incidence ratio

**Table 1(suite):** details of 23 effective coverage indicators included in the universal health coverage effective coverage index, by health service type, 2019 (source lancet 2020, ensuring universal health coverage, 1990-2019: by GBD universal health coverage collaborators)

Children younger than 5 years	Effective coverage indicator	Metric	Effective coverage indicator measurement	
			**Numerator**	**Denominator**
Treatment, communicable diseases and MNCH	Diarrhea treatment	MIR	Mortality from diarrheal diseases for children younger than 5 years	Incidence of diarrheal diseases for children younger than 5 years
Treatment, NCDs	Acute lymphoid leukemia treatment	MIR	Mortality from acute lymphoid leukemia for children aged 1-4 years	Incidence of acute lymphoid leukemia for children aged 1-4 years
**Children and adolescents (5-19 years)**				
Treatment, communicable diseases,MNCH	ART coverage	Coverage	Populations aged 5-19 years living with HIV/AIDS and on ART	Populations aged 5-19 years living with HIV/AIDS
Treatment, NCDs	Acute lymphoid leukemia treatment	MIR	Mortality from acute lymphoid leukemia for populations aged 5-19 years	Incidence of acute lymphoid leukemia for populations aged 5-19 years
Treatment, NCDs	Asthma treatment	MPR	Mortality from asthma for populations aged 5-19 years	Prevalence of asthma for populations aged 5-19 years
Treatment, NCDs	Epilepsy treatment	MPR	Mortality from epilepsy for populations aged 5-19 years	Prevalence of epilepsy for populations aged 5-19
Treatment, NCDs	Appendicitis treatment	MIR	Mortality from appendicitis for populations aged 5-19 years	Incidence of appendicitis for populations aged 5-19 years
Treatment, NCDs	Paralytic ileus and intestinal obstruction treatment	MIR	Mortality from paralytic ileus and intestinal obstruction for populations aged 5-19 years	Incidence of paralytic ileus and intestinal obstruction Populations aged 5-19 years

GBD= global burden of disease, NCDs= non-communicable diseases, MIR= mortality-to-incidence ratio, MPR= mortality-to-prevalence ratio, MNCH= maternal, neonatal, and child health, ART= antiretroviral therapy

**Table 1(suite 1):** details of 23 effective coverage indicators included in the universal health coverage effective coverage index, by health service type, 2019 (source lancet 2020, ensuring universal health coverage, 1990-2019: by GBD universal health coverage collaborators)

	Effective coverage indicator	Metric	Effective coverage indicator measurement	
			**Numerator**	**Denominator**
**Adults (20-64 years)**				
Treatment, communicable diseases and MNCH	ART coverage	Coverage	Population aged 20-64 years living with HIV/AIDS and on ART	Population aged 20-64 years living with HIV/AIDS
Treatment, communicable diseases and MNCH	Tuberculosis treatment	MIR	Mortality from tuberculosis for populations aged 20-64 years	Incidence of tuberculosis for populations aged 20-64 years
Treatment, NCDs	Diabetes treatment	MPR	Mortality from diabetes for populations aged 20-64 years	Prevalence of diabetes for populations aged 20-64 years
Treatment, NCDs	IHD treatment	RSDR	Risk-standardised deaths from IHD for populations aged 20-64 years	Population aged 20-64 years
Treatment, NCDs	Stroke treatment	MIR	Mortality from stroke for populations aged 20-64 years	Incidence of stroke for populations aged 20-64 years
Treatment, NCDs	CKD treatment	MPR	Mortality from CKD for populations aged 20-64 years	Incidence of CKD for populations aged 20-64 years
Treatment, NCDs	COPD treatment	MPR	Mortality from COPD for populations aged 20-64 years	Prevalence of COPD for populations aged 20-64 years
Treatment, NCDs	Cervical cancer treatment	MIR	Mortality from cervical cancer for females aged 20-64 years	Incidence of cervical cancer for females aged 20-64 years

GBD= global burden of disease, MNCH= maternal, neonatal, and child health, MIR= mortality-to-incidence ratio, NCDs= non-communicable diseases, ART= antiretroviral therapy, MPR= mortality-to-prevalence ratio, IHD= ischaemic heart disease, RSDR= risk-standardised death rate, CKD= chronic kidney disease, COPD= chronic obstructive pulmonary disease

**Table 1(suite 2):** details of 23 effective coverage indicators included in the universal health coverage effective coverage index, by health service type, 2019 (source lancet 2020, ensuring universal health coverage, 1990-2019: by GBD universal health coverage collaborators)

	Effective coverage indicator	Metric	Effective coverage indicator measurement	
			**Numerator**	**Denominator**
Treatment, NCDs	Breast cancer treatment	MIR	Mortality from breast cancer for females aged 20-64 years	Incidence of breast cancer for females aged 20-64 years
Treatment, NCDs	Uterine cancer treatment	MIR	Mortality from uterine cancer for females aged 20-64 years	Incidence of uterine cancer for females aged 20-64 years
Treatment, NCDs	Colon/rectum cancer treatment	MIR	Mortality from colon/rectum cancer for populations aged 20-64 years	Incidence of colon/rectum for populations aged 20-64 years
Treatment, NCDs	Epilepsy treatment	MPR	Mortality from epilepsy for populations aged 20-64 years	Prevalence of epilepsy for populations aged 20-64 years
Treatment, NCDs	Appendicitis treatment	MIR	Mortality from appendicitis for populations aged 20-64 years	Incidence of appendicitis for populations aged 20-64 years
Treatment, NCDs	Paralytic ileus and intestinal obstruction treatment	MIR	Mortality from paralytic ileus and intestinal obstruction for populations aged 20-64 years	Incidence of paralytic ileus and intestinal obstruction for populations aged 20-64 years
**Older adults (**≥ **65 years)**				
Treatment, communicable diseases and MNCH	ART coverage	Coverage	Population aged ≥65 years living with HIV/AIDS and on ART	Population aged ≥65 years living with HIV/AIDS
Treatment, communicable diseases and MNCH	Tuberculosis treatment	MIR	Mortality from tuberculosis for populations aged ≥65 years	Incidence of tuberculosis for populations aged ≥65 years
Treatment, NCDs	Diabetes treatment	MPR	Mortality from diabetes for populations aged ≥65 years	Prevalence of diabetes for populations aged ≥65 years

GBD= global burden of disease, NCDs= non-communicable diseases, MIR= mortality-to-incidence ratio, MPR= mortality-to-prevalence ratio, MNCH= maternal, neonatal, and child health, ART= antiretroviral therapy

**Table 1(suite 3):** details of 23 effective coverage indicators included in the universal health coverage effective coverage index, by health service type, 2019 (source lancet 2020, ensuring universal health coverage, 1990-2019: by GBD universal health coverage collaborators)

	Effective coverage indicator	Metric	Effective coverage indicator measurement	
			**Numerator**	**Denominator**
Treatment, NCDs	IHD treatment	RSDR	Risk-standardised deaths from IHD for populations aged ≥65 years	Population aged ≥65 years
Treatment, NCDs	Stroke treatment	MIR	Mortality from stroke for populations aged ≥65 years	Incidence of stroke aged ≥65 years
Treatment, NCDs	CKD treatment	MPR	Mortality from CKD for populations aged ≥65 years	Incidence of CKD aged ≥65 years
Treatment, NCDs	Cervical cancer treatment	MIR	Mortality from cervical cancer for females aged ≥65 years	Incidence of cervical cancer for females aged ≥65 years
Treatment, NCDs	Breast cancer treatment	MIR	Mortality from breast cancer for females aged ≥65 years	Incidence of breast cancer for females aged ≥65 years
Treatment, NCDs	Uterine cancer treatment	MIR	Mortality from uterine cancer for females aged ≥65 years	Incidence of uterine cancer for females aged ≥65 years
Treatment, NCDs	Colon/rectum cancer treatment	MIR	Mortality from colon/rectum cancer for populations aged ≥65 years	Incidence of colon/rectum cancer aged ≥65 years
Treatment, NCDs	Epilepsy treatment	MPR	Mortality from epilepsy for populations aged ≥65 years	Prevalence of epilepsy aged ≥65 years
Treatment, NCDs	Appendicitis treatment	MIR	Mortality from appendicitis for populations aged ≥65 years	Incidence of appendicitis aged ≥65 years
Treatment, NCDs	Paralytic ileus and intestinal obstruction treatment	MIR	Mortality from paralytic ileus and intestinal obstruction for populations aged ≥65 years	Incidence of paralytic ileus and intestinal obstruction aged ≥65 years

GBD= global burden of disease, NCDs= non-communicable diseases, MIR= mortality-to-incidence ratio, MPR= mortality-to-prevalence ratio, RSDR= risk-standardised death rate, CKD= chronic kidney disease, IHD= ischaemic heart disease

**Review of national and strategic plan achievements towards sustainable development goals:** we reviewed the common country analysis, an independent and collective review by the UN Country Team on the progress of Tanzania towards the 2030 Agenda across all SDG targets and the UN norms and standards. We used the internal Strengths and Weaknesses and external Opportunities and Threats (SWOT) analysis for the assessment findings. We reviewed the country´s progress in the triple billion targets stated in the GPW13. Since the GPW13 covers a 5-year plan (2019-2023), we used the end-of-biennium report for 2020 and 2021 that highlights the status of the various outputs selected by the country as a measure of progress in achieving the health-related SDGs in the country. The end of the biennium assessment analysed the achievements of technical support and enabling functions of WHO to the member state using the various outputs monitored across the biennium. Each of the outputs was analysed in detail across six dimensions: technical support, leadership, global goods or technical products, Gender Equity, Human Rights and Disability (GEHRD), value for money and results. We reviewed the outputs of the analysis to identify areas of strength and weakness in achieving the outputs. We reviewed the national health sector strategic plan for Tanzania Mainland and Zanzibar: our review focused on two areas: 1) the extent of alignment of the strategic priority areas with the global priorities of SDGs and GPW13; 2) the success and challenges in the achievements of the priority areas. We also reviewed the monitoring and evaluation framework for the national health sector strategic plan.

**Figure 2:**
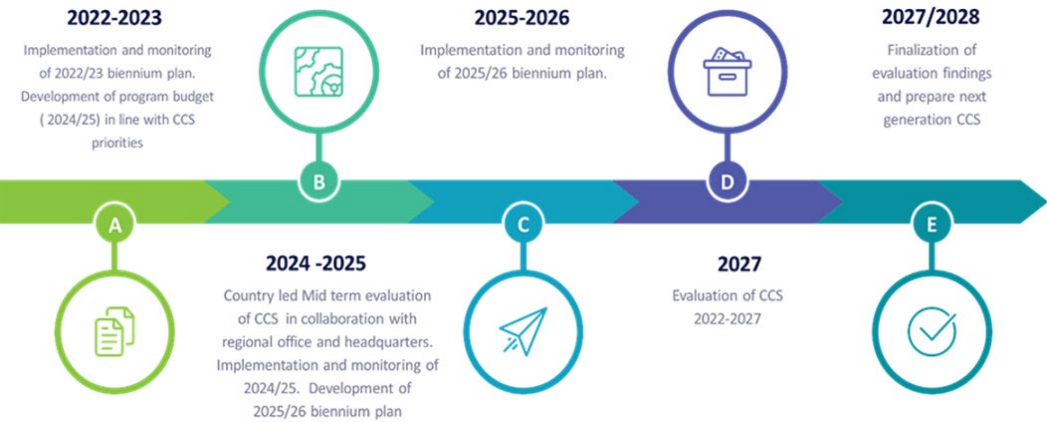
milestones in the monitoring and evaluation of WHO Country Cooperation Strategy 2022-2027, United Republic of Tanzania

**Stakeholder analysis and consultations:** we conducted stakeholder analysis and mapping exercises. After a brainstorming exercise in analysing the stakeholders, we mapped them based on key criteria on areas of collaboration to manage during the implementation of the CCS. We reviewed the literature on how a mix of stakeholders improves strategic planning. The reason behind this is those affected by decisions in the strategic plan priorities contribute to an effective process [18]. We had continuous engagement and consultations with the Tanzania Ministry of Health officials. We reviewed the available evidence and discussed the selection of critical priority areas and interventions that we should focus on to achieve results. In addition, a discussion was made on monitoring and evaluating the CCS document. We laid out a monitoring and evaluation framework for the CCS. We engaged all stakeholders to agree on the mechanism and timeline for monitoring the CCS (Figure 2).

**Results**

**Figure 3:**
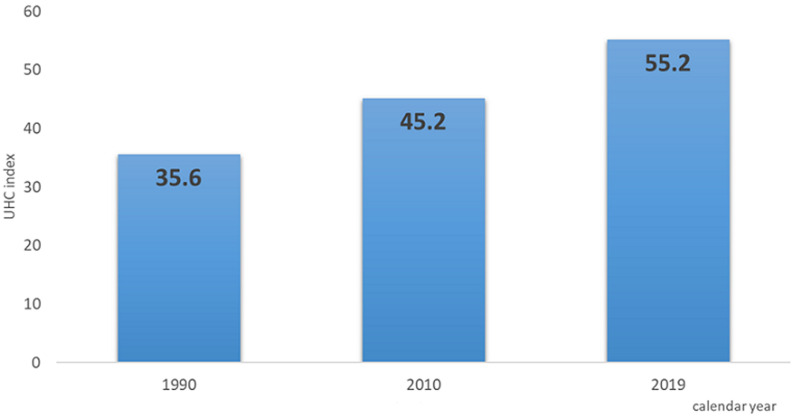
universal health coverage (UHC) index measuring the effectiveness of essential health service (measured on a scale from 0(worst) to 100 (best), 1990 to 2019, Tanzania (source global burden of disease (GBD))

The performance on the overall UHC effective coverage index that covers 23 indicators in the health system, on a scale of 0-100, for Tanzania improved from 45.2 in 2010 to 55.2 in 2019. While among the 23 indicators in the index, maternal and newborn care and non-communicable diseases scored below 20 (Figure 3). Estimated death from all causes showed a decline over time. In 2019, based on the latest Global Burden of Disease (GBD) estimate for Tanzania, it was 624.5 deaths per 100000 population. The global estimate for the same period was 730.5 per 100,000. Communicable, maternal, neonatal, and non-communicable diseases accounted for the country´s 10 top causes of death. The review of the common country assessment towards achieving SDGs identified 10 accelerators for Tanzania to be on track to the SDGs target. Four of the accelerators, addressing maternal health, reducing the adolescent birth rate, ending the epidemic of HIV, TB, and malaria, and addressing malnutrition amongst adolescent girls and young women and < 5-year-old children, were directly linked with health-related SDGs. The SWOT analysis of achieving the SDGs using the common country analysis revealed an improvement in geographical access to health services because of the increased construction of health facilities. In addition, outpatient department utilization per person per year increased to 0.85 in 2019 compared to 0.8 in 2016. The political commitment and availability of strong partnerships with the availability of strong strategic plans were observed as an opportunity for achieving health targets. The assessment revealed a maternal mortality rate of 556 per 10000 in 2015, which was higher than the 2010 figure. Only 50% of the target for availing health workers in health facilities was achieved.

The end of the biennium assessment for 2020/21 of WHO´s support for countries´ priorities on technical support has shown a gap in the inclusion of Gender, Equity, Human Rights, and Disability (GEHRD) in planning and implementation. Four strategic priorities were identified based on a detailed analysis of the country´s context. The identified priority areas are strengthening the health system, protecting health emergencies, reducing or controlling risk factors, and health governance, leadership, and accountability. A total of 13 intervention areas were identified for the four priority areas. The priority areas aligned with the priorities of the national strategic plan, the outcomes of the GPW13, the SDG targets, and the priority areas of the UN Sustainable Development Cooperation Framework for Tanzania (Table 2, Table 2 (suite)). A total of 25 Development Partners Group for Health (DPG-H) were mapped based on their area of focus for various health interventions. A total budget of >73 million US$ was proposed for implementing the CCS for 6 years; 59% of the budget is for health system strengthening, followed by protecting people from health emergencies of 23%. The remaining 18% of the budget is for preventing risk factors and governance and leadership functions. To support the country in achieving the national GPW 13 and health-related SDGs priorities in health, the WHO country office and stakeholders developed a 6-year CCS (CCS 2022-2027) midterm strategic document [19]. The strategic document articulated the support of the three levels of the organization (country office, regional and headquarters in WHO) to lead the partnership in the health agenda in the country.

**Table 2:** alignment of the CCS priorities with the national priorities, the GPW13, the UNSDCF, and the sustainable development goals

Strategic priority area	National strategic priorities/results	GPW13 outcome areas	UNSDCF 2022-2027	SDG targets
Strengthen health systems to ensure universal access to quality RMNCAH and essential health services	Strategic outcome; outcome 5.3.1, and 5.3.2 ensure availability of quality of essential health care services and interventions	Outcome 1.1,1.2,1.3: - improved access to quality health services, and reduce the number of people suffering financial burden	Outcome 1: people in the URT, especially the most vulnerable, increasingly utilize quality health service	3.8, 3.c: - achieve universal health coverage, including financial risk protection, 3.1,3.3 by 2030, reduce the global maternal mortality ratio to less than 70 per 100,000 live births, and end the epidemics of AIDS, TB, malaria and NTDs…
Communities are protected against public health emergencies	Outcome 5.1.7 a sufficient capacity to prepare, detect, prevent, respond to, and recover from health epidemics, emergencies, and disasters	Outcome 2.1,2.2,2.3 - countries prepared, prevented, detect, and respond to epidemics	Outcome 3: people in the URT, benefit from more inclusive and gender-responsive management of natural resources	3.d strengthen the capacity of all countries, for early warning, risk reduction and management of national and global health risks

CCS= country cooperation strategy, UNSDCF= United Nations sustainable development framework, SDGs= sustainable development goal, GPW13= 13^th^ global program of work (GPW13), RMNCAH= reproductive, maternal, newborn, child, and adolescent health, URT= United Republic of Tanzania, NTDs= neglected tropical diseases, TB= tuberculosis

**Table 2(suite):** alignment of the CCS priorities with the national priorities, the GPW13, the UNSDCF, and the sustainable development goals

Strategic priority area	National strategic priorities/results	GPW13 outcome areas	UNSDCF 2022-2027	SDG targets
Individuals are not exposed to risk factors threatening their health and well-being	Outcome 5.1.9: - health equity and well-being of the population irrespective of socio-economic status, gender, geographical and cultural diversity	Outcome 3.1, 3.2 and 3.2: - determinants of health addressed risk factors reduced	Outcome 3: people in the URT, benefit from more inclusive and gender-responsive management of natural resources, climate change resilience, disaster risk reduction	3.a strengthen the implementation of the WHO framework convention on tobacco control, 3.b promote mechanisms for raising capacity for effective climate change
Improved efficiencies in the health sector through better Health governance, leadership, and accountability	Strategic outcome 6.3: - a functioning governance structure in place aligned with government policies	Outcome 4.1: - strengthening country capacity in data and information; outcome 4.2: - strengthened leadership, governance	Outcome 1: people in the URT, especially the most vulnerable, increasingly utilize quality health service nutrition, and protection services	

CCS= country cooperation strategy, UNSDCF= United Nations sustainable development framework, SDGs= sustainable development goal, GPW13= 13^th^ global program of work (GPW13), RMNCAH= reproductive, maternal, newborn, child, and adolescent health, URT= United Republic of Tanzania

**Discussion**

A 6-year CCS was developed from 2022-2027 to support the United Republic of Tanzania towards achieving the health-related SDGs. This strategic document was aligned with the national health sector strategic plan, GPW13 and the SGDs. This alignment would help the WHO to support the country´s effort towards achieving health and health-related indicators. It is also aligned with the Tanzania United Nations Sustainable Development Cooperation Framework (UNSDCF 2022-2027). This would allow the WHO to discharge its responsibility of coordinating and leading the health intervention of the UN in the country. The availability of CCS 2022-2027 in the country office organizes the way WHO brings all health stakeholders in the country, and it also elaborates on the responsibility and accountability of all actors and clarifies priority areas, interventions, and indicators of success across time during the implementation period of the strategic plan. Hence, it facilitates collaboration and avoids duplications of efforts by encouraging value for money. This is in line with studies that confirm the analysis of alignment in the strategic plan by vision, mission and values across stakeholders found to be effective in delivering the desired outcomes. The key to ensuring country strategic plans, by extension, SDG goals, can only be achieved through multi-stakeholder partnerships and engagement [20,21].

We noted stakeholders´ skills, resources, leadership, and broader participation as a marker of success in the strategic plan. This is in line with the findings of C. Bloom stated that what contributes to the success of strategic plans. The situation analysis, use of evaluation findings in the health sector, and involvement of all stakeholders, including the leadership in WHO and the Ministry of Health, are best practices in developing the CCS that could be cited as a potential indicator for the success of the implementation of the strategic plan [22]. Though an assessment was done in the previous CCS implementation, an in-depth evaluation is lacking, which limits the availability of clear evaluation recommendations to be reflected in the current CCS. However, we reviewed the assessment and did a desk review of the previous CCS considering current health indicators to address issues that were not implemented.

## Conclusion

The persistent involvement of stakeholders and the discussion to focus on priority areas and interventions for the 6 years of CCS in line with the national and global priorities helped to develop the CCS. We believe the process we employed will lead to having detailed operational plans for implementation for achieving SDG targets. We recommend developing a detailed business case for resource mobilization and charting the road for implementation. It is also essential to focus on monitoring performance and regular feedback mechanisms to ensure the investment in developing the strategic plan is in good use.

### What is known about this topic

- Alignment of the various strategic visions, missions and priorities is crucial for the success of strategic plans;
- Stakeholder engagement and participatory approach right from the inception of the concept in strategic plan to the finalization of the plan is critical in realization of goals and objectives as well as underlining accountability of each actor in the plan;
- The CCS has been a midterm strategic vision for WHO to support member states.

### What this study adds

- The methods that we used in meticulous review of documents in the prioritization of interventions and focus areas, and the mapping of various strategic priority areas for alignment would help countries that will undergo similar process to follow. In addition, this helped in aligning the monitoring and evaluation framework of the CCS with the UNSDCF and the national health sector strategic plan.
